# μ_4_-Sulfido-bis­{(μ-2-furyl­methane­thiol­ato)bis­[tricarbonyl­iron](*Fe*—*Fe*)}

**DOI:** 10.1107/S1600536810022634

**Published:** 2010-06-18

**Authors:** Cong Han, Xiao-Nan Huang, Yan-Ling Guo, Wei Liu

**Affiliations:** a College of Sciences, Tianjin University of Science and Technology, Tianjin 300457, People’s Republic of China

## Abstract

The title compound, [Fe_4_(C_5_H_5_OS)_2_S(CO)_12_], was prepared by the direct reaction of Fe_3_(CO)_12_ and 2-furyl­methane­thiol in tetra­hydro­furan. Desulfurization took place readily to form an Fe_4_S_3_ cluster. The mol­ecule consists of two similar [(μ-2-C_4_H_3_O—CH_2_S)Fe_2_(CO)_6_] moieties joined to a spiro-type four-coordinate μ_4_-S atom such that this bridging sulfur is tetra­hedrally coordinated to the four Fe atoms. In each diiron subcluster core, the 2-furyl­methane­thiol­ate ligand bridges the two Fe atoms.

## Related literature

For related cluster complexes with a spiro-type μ_4_-S atom which is tetrahedrally coordinated to the four iron atoms, see: Coleman *et al.* (1967 ▶); Shaver *et al.*, (1979 ▶). For Fe—Fe bond lengths in related structures, see: Song *et al.* (1988 ▶, 1991 ▶, 1992 ▶).
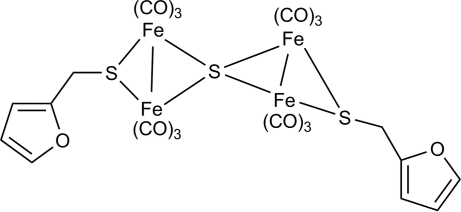


## Experimental

### 

#### Crystal data

[Fe_4_(C_5_H_5_OS)_2_S(CO)_12_]
*M* *_r_* = 817.91
Triclinic, 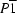

*a* = 9.0335 (18) Å
*b* = 9.984 (2) Å
*c* = 16.821 (3) Å
α = 82.89 (3)°
β = 75.75 (3)°
γ = 89.68 (3)°
*V* = 1458.6 (5) Å^3^
*Z* = 2
Mo *K*α radiation
μ = 2.23 mm^−1^
*T* = 113 K
0.24 × 0.20 × 0.16 mm

#### Data collection

Bruker SMART APEX CCD area-detector diffractometer
Absorption correction: multi-scan (*SADABS*; Sheldrick, 1996 ▶) *T*_min_ = 0.539, *T*_max_ = 0.697
10642 measured reflections
5099 independent reflections
4055 reflections with *I* > 2σ(*I*)
*R*_int_ = 0.030

#### Refinement

*R*[*F* ^2^ > 2σ(*F* ^2^)] = 0.031
*wR*(*F* ^2^) = 0.078
*S* = 1.02
5099 reflections
388 parameters
H-atom parameters constrained
Δρ_max_ = 0.45 e Å^−3^
Δρ_min_ = −0.62 e Å^−3^

### 

Data collection: *SMART* (Bruker, 1999 ▶); cell refinement: *SAINT* (Bruker, 1999 ▶); data reduction: *SAINT*; program(s) used to solve structure: *SHELXS97* (Sheldrick, 2008 ▶); program(s) used to refine structure: *SHELXL97* (Sheldrick, 2008 ▶); molecular graphics: *SHELXTL* (Sheldrick, 2008 ▶); software used to prepare material for publication: *SHELXTL*.

## Supplementary Material

Crystal structure: contains datablocks I, global. DOI: 10.1107/S1600536810022634/jh2165sup1.cif

Structure factors: contains datablocks I. DOI: 10.1107/S1600536810022634/jh2165Isup2.hkl

Additional supplementary materials:  crystallographic information; 3D view; checkCIF report

## Figures and Tables

**Fig. 1.**
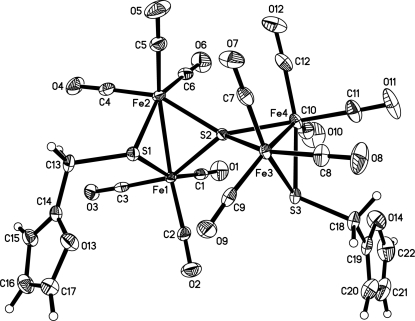
The structure of (I), with displacement ellipsoids for non-H atoms drawn at the 50% probability level.

**Fig. 2.**
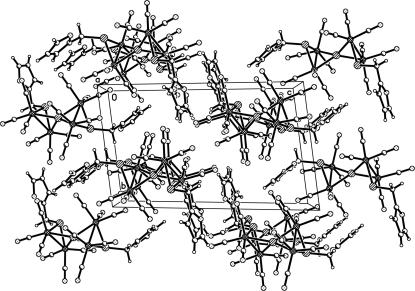
A packing diagram for (I).

## supplementary crystallographic information

### Comment

In the title compound (Fig. 1), the molecule consists of two identical [(µ-2-C_4_H_3_O—CH_2_S)Fe_2_(CO)_6_] moieties joined to a spiro-type four-coordinate µ_4_-S atom, which is situated at the center of a distorted tetrahedron made up of four iron atoms. So that this bridging sulfur is tetrahedrally coordinated to the four iron atoms. The four S—Fe bond lengths around the central S atom are 2.2404 (11), 2.2411 (9), 2.2451 (10) and 2.2354 (9) Å. The two Fe—Fe bond distances (average, 2.532 Å) are shorter than the average Fe—Fe bond distance (2.540 Å) in [(µ-EtS)Fe_2_(CO)_6_]2(µ_4_-S) (Song *et al.*, 1988), but slightly longer than the average Fe—Fe distance (2.523 Å) both in [(µ-EtS)Fe_2_(CO)_6_](µ_4_-S)[µ-PhS)Fe_2_(CO)_6_] (Song *et al.*, 1991) and in [(µ-nBuS)Fe_2_(CO)_6_](µ_4_-S) [(µ-PhS Fe_2_(CO)_6_] (Song *et al.*, 1992). However, the bond lengths between the four iron atoms and two sulfur atoms are very close to each other [Fe(1)—S(1) 2.2608 (9), Fe(2)—S(1) 2.2721 (11), Fe(3)—S(3) 2.2623 (9), Fe(4)—S(3) 2.2683 Å]. The bond angles of Fe(1)—S(2)—Fe(2) and Fe(4)—S(2)—Fe(3) are 68.62 (3)° and 69.00 (3)° respectively, which represents a distorted tetrahedron geometry around the central S(2) atom. In addition, it can be seen intuitively from Fig. 1 that each Fe atom has three terminal CO ligands, and the two 2-furfuryl groups attached to the cluster core Fe_4_S_3_ are both bonded to µ-S(1) and µ-S(2) by an e-type of bond (Shaver *et al.*, 1979).

### Experimental

Fe_3_(CO)_12_ (1.00 g, 2 mmol) was added to a solution of 2-furylmethanethiol (0.54 g, 2 mmol) in THF (20 ml). After the solution was stirred at room temperature for 12 h, the solvent was removed under reduced pressure and the residue was chromatographed on a silica gel column with CH_2_Cl_2_/petroleum ether (1:20 *v*/*v*) as the eluent to give the title compound as a red solid (yield 19%, 0.31 g). Single crystals of the title compound were obtained by slow diffusion of petroleum ether into a solution of the complex in CH_2_Cl_2_ at room temperature.

### Refinement

The H atoms were included in calculated positions and refined using a riding model approximation. Constrained C—H bond lengths and isotropic U parameters: 0.95 Å and *U*_iso_(H) = 1.2*U*_eq_(C) for C*sp*^2^—H; 0.99 Å and *U*_iso_(H) = 1.2*U*_eq_(C) for methylene C—H

### Crystal data

**Table:**

[Fe_4_(C_5_H_5_OS)_2_S(CO)_12_]	*Z* = 2
*M**_r_* = 817.91	*F*(000) = 812
Triclinic, *P*1	*D*_x_ = 1.862 Mg m^−^^3^
Hall symbol: -P 1	Mo *K*α radiation, λ = 0.71073 Å
*a* = 9.0335 (18) Å	Cell parameters from 4907 reflections
*b* = 9.984 (2) Å	θ = 2.1–27.9°
*c* = 16.821 (3) Å	µ = 2.23 mm^−^^1^
α = 82.89 (3)°	*T* = 113 K
β = 75.75 (3)°	Block, red
γ = 89.68 (3)°	0.24 × 0.20 × 0.16 mm
*V* = 1458.6 (5) Å^3^	

### Data collection

**Table:**

Bruker SMART APEX CCD area-detector diffractometer	5099 independent reflections
Radiation source: fine-focus sealed tube	4055 reflections with *I* > 2σ(*I*)
graphite	*R*_int_ = 0.030
φ and ω scans	θ_max_ = 25.0°, θ_min_ = 2.1°
Absorption correction: multi-scan (*SADABS*; Sheldrick, 1996)	*h* = −7→10
*T*_min_ = 0.539, *T*_max_ = 0.697	*k* = −11→11
10642 measured reflections	*l* = −19→19

### Refinement

**Table:**

Refinement on *F*^2^	Primary atom site location: structure-invariant direct methods
Least-squares matrix: full	Secondary atom site location: difference Fourier map
*R*[*F*^2^ > 2σ(*F*^2^)] = 0.031	Hydrogen site location: inferred from neighbouring sites
*wR*(*F*^2^) = 0.078	H-atom parameters constrained
*S* = 1.02	*w* = 1/[σ^2^(*F*_o_^2^) + (0.045*P*)^2^] where *P* = (*F*_o_^2^ + 2*F*_c_^2^)/3
5099 reflections	(Δ/σ)_max_ = 0.001
388 parameters	Δρ_max_ = 0.45 e Å^−^^3^
0 restraints	Δρ_min_ = −0.62 e Å^−^^3^

### Special details

**Table:**

Geometry. All e.s.d.'s (except the e.s.d. in the dihedral angle between two l.s. planes) are estimated using the full covariance matrix. The cell e.s.d.'s are taken into account individually in the estimation of e.s.d.'s in distances, angles and torsion angles; correlations between e.s.d.'s in cell parameters are only used when they are defined by crystal symmetry. An approximate (isotropic) treatment of cell e.s.d.'s is used for estimating e.s.d.'s involving l.s. planes.
Refinement. Refinement of *F*^2^ against ALL reflections. The weighted *R*-factor *wR* and goodness of fit *S* are based on *F*^2^, conventional *R*-factors *R* are based on *F*, with *F* set to zero for negative *F*^2^. The threshold expression of *F*^2^ > σ(*F*^2^) is used only for calculating *R*-factors(gt) *etc*. and is not relevant to the choice of reflections for refinement. *R*-factors based on *F*^2^ are statistically about twice as large as those based on *F*, and *R*- factors based on ALL data will be even larger.

### Fractional atomic coordinates and isotropic or equivalent isotropic displacement parameters (Å^2^)

**Table:**

	*x*	*y*	*z*	*U*_iso_*/*U*_eq_	
Fe1	0.19683 (4)	0.85862 (4)	0.10808 (2)	0.01296 (11)	
Fe2	−0.01371 (4)	0.67940 (4)	0.14885 (2)	0.01566 (11)	
Fe3	0.28968 (4)	0.61770 (4)	0.29734 (2)	0.01501 (11)	
Fe4	0.08366 (5)	0.77162 (4)	0.36367 (2)	0.01835 (12)	
S1	0.22855 (8)	0.64471 (7)	0.07704 (4)	0.01665 (16)	
S2	0.13073 (7)	0.73270 (7)	0.23184 (4)	0.01353 (15)	
S3	0.33321 (8)	0.83480 (7)	0.31273 (4)	0.01675 (16)	
O1	0.0447 (3)	1.0895 (2)	0.18284 (14)	0.0338 (6)	
O2	0.5239 (2)	0.9209 (2)	0.08740 (14)	0.0345 (6)	
O3	0.1571 (2)	0.9649 (2)	−0.05598 (12)	0.0250 (5)	
O4	−0.1291 (3)	0.7242 (2)	0.00010 (14)	0.0371 (6)	
O5	−0.1418 (3)	0.4047 (3)	0.21532 (16)	0.0494 (7)	
O6	−0.2606 (3)	0.8400 (3)	0.23400 (15)	0.0416 (6)	
O7	0.1210 (2)	0.3632 (2)	0.30600 (14)	0.0349 (6)	
O8	0.4142 (3)	0.5177 (2)	0.43804 (15)	0.0447 (7)	
O9	0.5642 (2)	0.5837 (2)	0.16440 (13)	0.0301 (5)	
O10	−0.0364 (3)	1.0434 (3)	0.38395 (17)	0.0513 (7)	
O11	0.1137 (3)	0.6883 (3)	0.53228 (14)	0.0544 (8)	
O12	−0.2036 (3)	0.6167 (3)	0.39323 (15)	0.0509 (7)	
O13	0.5272 (2)	0.6459 (2)	−0.06799 (12)	0.0268 (5)	
O14	0.3290 (2)	1.0632 (2)	0.43411 (14)	0.0341 (6)	
C1	0.1063 (3)	1.0016 (3)	0.15260 (18)	0.0191 (6)	
C2	0.3963 (3)	0.9011 (3)	0.09646 (17)	0.0197 (6)	
C3	0.1750 (3)	0.9236 (3)	0.00758 (18)	0.0168 (6)	
C4	−0.0849 (3)	0.7046 (3)	0.05842 (19)	0.0232 (7)	
C5	−0.0906 (3)	0.5097 (3)	0.19004 (19)	0.0256 (7)	
C6	−0.1661 (3)	0.7747 (3)	0.20174 (18)	0.0243 (7)	
C7	0.1872 (3)	0.4617 (3)	0.30277 (17)	0.0221 (7)	
C8	0.3660 (4)	0.5558 (3)	0.38300 (19)	0.0258 (7)	
C9	0.4556 (3)	0.5937 (3)	0.21485 (18)	0.0210 (7)	
C10	0.0084 (4)	0.9395 (3)	0.3745 (2)	0.0294 (7)	
C11	0.1017 (4)	0.7239 (4)	0.4665 (2)	0.0326 (8)	
C12	−0.0913 (4)	0.6772 (3)	0.38333 (18)	0.0302 (8)	
C13	0.2498 (3)	0.6287 (3)	−0.03314 (17)	0.0247 (7)	
H13A	0.2449	0.5318	−0.0399	0.030*	
H13B	0.1634	0.6731	−0.0514	0.030*	
C14	0.3945 (3)	0.6892 (3)	−0.08635 (17)	0.0199 (6)	
C15	0.4244 (3)	0.7765 (3)	−0.15620 (18)	0.0246 (7)	
H15	0.3517	0.8210	−0.1818	0.030*	
C16	0.5855 (4)	0.7893 (3)	−0.18430 (19)	0.0281 (7)	
H16	0.6417	0.8434	−0.2326	0.034*	
C17	0.6433 (4)	0.7104 (3)	−0.12963 (19)	0.0283 (7)	
H17	0.7492	0.7003	−0.1329	0.034*	
C18	0.4375 (3)	0.8527 (3)	0.39289 (18)	0.0231 (7)	
H18A	0.3794	0.8061	0.4469	0.028*	
H18B	0.5386	0.8110	0.3782	0.028*	
C19	0.4570 (3)	0.9976 (3)	0.39882 (17)	0.0232 (7)	
C20	0.5770 (4)	1.0843 (4)	0.3741 (2)	0.0353 (8)	
H20	0.6785	1.0643	0.3470	0.042*	
C21	0.5229 (5)	1.2123 (4)	0.3963 (2)	0.0397 (9)	
H21	0.5812	1.2941	0.3873	0.048*	
C22	0.3763 (5)	1.1948 (3)	0.4318 (2)	0.0417 (9)	
H22	0.3113	1.2640	0.4530	0.050*	

### Atomic displacement parameters (Å^2^)

**Table:**

	*U*^11^	*U*^22^	*U*^33^	*U*^12^	*U*^13^	*U*^23^
Fe1	0.0125 (2)	0.0137 (2)	0.0129 (2)	−0.00174 (16)	−0.00465 (15)	0.00090 (16)
Fe2	0.0133 (2)	0.0175 (2)	0.0165 (2)	−0.00366 (16)	−0.00510 (16)	−0.00030 (17)
Fe3	0.0178 (2)	0.0158 (2)	0.0117 (2)	0.00372 (17)	−0.00451 (16)	−0.00106 (16)
Fe4	0.0193 (2)	0.0215 (2)	0.0120 (2)	0.00411 (18)	0.00028 (17)	−0.00232 (18)
S1	0.0182 (3)	0.0168 (4)	0.0150 (4)	−0.0004 (3)	−0.0036 (3)	−0.0031 (3)
S2	0.0123 (3)	0.0157 (3)	0.0121 (3)	0.0000 (3)	−0.0028 (3)	−0.0001 (3)
S3	0.0206 (4)	0.0186 (4)	0.0134 (4)	0.0025 (3)	−0.0081 (3)	−0.0031 (3)
O1	0.0421 (14)	0.0217 (12)	0.0389 (14)	0.0091 (11)	−0.0105 (11)	−0.0089 (11)
O2	0.0217 (12)	0.0459 (15)	0.0353 (14)	−0.0092 (10)	−0.0113 (10)	0.0055 (11)
O3	0.0239 (11)	0.0332 (12)	0.0179 (11)	0.0018 (10)	−0.0098 (9)	0.0056 (10)
O4	0.0402 (14)	0.0438 (15)	0.0330 (14)	−0.0125 (12)	−0.0244 (12)	0.0051 (11)
O5	0.0608 (17)	0.0338 (15)	0.0575 (17)	−0.0266 (13)	−0.0323 (14)	0.0168 (13)
O6	0.0250 (12)	0.0576 (17)	0.0409 (15)	0.0117 (12)	−0.0028 (11)	−0.0123 (13)
O7	0.0308 (12)	0.0259 (13)	0.0413 (15)	−0.0053 (11)	0.0023 (11)	−0.0011 (11)
O8	0.0757 (19)	0.0317 (14)	0.0387 (15)	0.0098 (13)	−0.0388 (14)	−0.0010 (12)
O9	0.0185 (11)	0.0395 (14)	0.0313 (13)	0.0022 (10)	0.0008 (10)	−0.0151 (11)
O10	0.0561 (17)	0.0366 (16)	0.0624 (19)	0.0236 (14)	−0.0121 (14)	−0.0166 (14)
O11	0.0680 (19)	0.074 (2)	0.0149 (14)	0.0192 (16)	−0.0027 (12)	0.0020 (13)
O12	0.0365 (15)	0.0602 (18)	0.0447 (16)	−0.0180 (14)	0.0077 (12)	0.0013 (14)
O13	0.0256 (11)	0.0307 (12)	0.0205 (11)	0.0003 (10)	−0.0013 (9)	0.0014 (10)
O14	0.0369 (13)	0.0310 (13)	0.0323 (13)	0.0081 (11)	−0.0011 (11)	−0.0110 (11)
C1	0.0163 (14)	0.0192 (15)	0.0211 (16)	−0.0049 (12)	−0.0062 (12)	0.0036 (13)
C2	0.0217 (16)	0.0216 (15)	0.0169 (15)	−0.0044 (12)	−0.0086 (12)	0.0016 (12)
C3	0.0101 (13)	0.0164 (14)	0.0236 (17)	−0.0022 (11)	−0.0038 (12)	−0.0015 (13)
C4	0.0204 (15)	0.0233 (16)	0.0257 (18)	−0.0070 (13)	−0.0062 (13)	−0.0014 (14)
C5	0.0231 (16)	0.0294 (18)	0.0248 (17)	−0.0068 (14)	−0.0099 (13)	0.0026 (14)
C6	0.0146 (15)	0.0352 (18)	0.0219 (16)	−0.0068 (14)	−0.0029 (13)	−0.0022 (14)
C7	0.0254 (16)	0.0212 (16)	0.0172 (15)	0.0063 (14)	−0.0020 (12)	0.0012 (13)
C8	0.0360 (18)	0.0209 (16)	0.0235 (18)	0.0062 (14)	−0.0121 (15)	−0.0048 (14)
C9	0.0229 (16)	0.0214 (16)	0.0243 (17)	0.0044 (13)	−0.0143 (14)	−0.0080 (13)
C10	0.0280 (17)	0.032 (2)	0.0273 (18)	0.0078 (15)	−0.0049 (14)	−0.0064 (15)
C11	0.0361 (19)	0.041 (2)	0.0181 (18)	0.0111 (16)	−0.0010 (14)	−0.0061 (16)
C12	0.0297 (18)	0.0357 (19)	0.0186 (17)	0.0010 (16)	0.0050 (14)	−0.0010 (14)
C13	0.0314 (17)	0.0284 (17)	0.0166 (15)	−0.0054 (14)	−0.0068 (13)	−0.0092 (13)
C14	0.0221 (15)	0.0214 (15)	0.0164 (15)	−0.0009 (12)	−0.0019 (12)	−0.0087 (13)
C15	0.0236 (16)	0.0315 (17)	0.0177 (16)	0.0038 (13)	−0.0044 (13)	−0.0007 (13)
C16	0.0374 (19)	0.0234 (17)	0.0193 (16)	−0.0023 (15)	−0.0003 (14)	0.0006 (14)
C17	0.0246 (16)	0.0326 (18)	0.0264 (18)	−0.0014 (14)	−0.0029 (14)	−0.0054 (15)
C18	0.0287 (16)	0.0260 (17)	0.0194 (16)	0.0041 (14)	−0.0136 (13)	−0.0059 (13)
C19	0.0266 (16)	0.0307 (17)	0.0176 (16)	0.0064 (14)	−0.0131 (13)	−0.0084 (14)
C20	0.0357 (19)	0.039 (2)	0.038 (2)	0.0031 (16)	−0.0203 (16)	−0.0060 (17)
C21	0.059 (2)	0.0292 (19)	0.040 (2)	−0.0040 (18)	−0.0297 (19)	−0.0040 (16)
C22	0.068 (3)	0.0255 (19)	0.035 (2)	0.0100 (18)	−0.0161 (19)	−0.0111 (16)

### Geometric parameters (Å, °)

**Table:**

Fe1—C3	1.791 (3)	O6—C6	1.141 (4)
Fe1—C1	1.793 (3)	O7—C7	1.142 (3)
Fe1—C2	1.811 (3)	O8—C8	1.141 (4)
Fe1—S2	2.2404 (11)	O9—C9	1.141 (3)
Fe1—S1	2.2608 (9)	O10—C10	1.127 (4)
Fe1—Fe2	2.5263 (10)	O11—C11	1.151 (4)
Fe2—C4	1.782 (3)	O12—C12	1.149 (4)
Fe2—C6	1.785 (3)	O13—C14	1.364 (4)
Fe2—C5	1.822 (3)	O13—C17	1.377 (4)
Fe2—S2	2.2411 (9)	O14—C19	1.367 (4)
Fe2—S1	2.2721 (11)	O14—C22	1.376 (4)
Fe3—C8	1.788 (3)	C13—C14	1.474 (4)
Fe3—C7	1.798 (3)	C13—H13A	0.9900
Fe3—C9	1.812 (3)	C13—H13B	0.9900
Fe3—S2	2.2451 (10)	C14—C15	1.344 (4)
Fe3—S3	2.2623 (9)	C15—C16	1.416 (4)
Fe3—Fe4	2.5377 (10)	C15—H15	0.9500
Fe4—C12	1.783 (3)	C16—C17	1.338 (5)
Fe4—C11	1.783 (3)	C16—H16	0.9500
Fe4—C10	1.818 (3)	C17—H17	0.9500
Fe4—S2	2.2354 (9)	C18—C19	1.476 (4)
Fe4—S3	2.2683 (10)	C18—H18A	0.9900
S1—C13	1.843 (3)	C18—H18B	0.9900
S3—C18	1.850 (3)	C19—C20	1.341 (4)
O1—C1	1.142 (3)	C20—C21	1.426 (5)
O2—C2	1.140 (3)	C20—H20	0.9500
O3—C3	1.147 (3)	C21—C22	1.315 (5)
O4—C4	1.142 (4)	C21—H21	0.9500
O5—C5	1.137 (4)	C22—H22	0.9500
			
C3—Fe1—C1	92.11 (13)	C13—S1—Fe2	114.86 (11)
C3—Fe1—C2	100.43 (13)	Fe1—S1—Fe2	67.74 (4)
C1—Fe1—C2	101.10 (13)	Fe4—S2—Fe1	135.87 (4)
C3—Fe1—S2	155.11 (8)	Fe4—S2—Fe2	134.78 (4)
C1—Fe1—S2	90.34 (10)	Fe1—S2—Fe2	68.62 (3)
C2—Fe1—S2	103.38 (10)	Fe4—S2—Fe3	69.00 (3)
C3—Fe1—S1	93.47 (9)	Fe1—S2—Fe3	126.33 (4)
C1—Fe1—S1	159.33 (9)	Fe2—S2—Fe3	134.36 (4)
C2—Fe1—S1	97.44 (10)	C18—S3—Fe3	113.61 (10)
S2—Fe1—S1	76.45 (4)	C18—S3—Fe4	114.22 (10)
C3—Fe1—Fe2	99.71 (9)	Fe3—S3—Fe4	68.13 (4)
C1—Fe1—Fe2	103.08 (9)	C14—O13—C17	105.9 (2)
C2—Fe1—Fe2	147.72 (10)	C19—O14—C22	105.5 (3)
S2—Fe1—Fe2	55.70 (3)	O1—C1—Fe1	177.5 (3)
S1—Fe1—Fe2	56.34 (3)	O2—C2—Fe1	176.0 (3)
C4—Fe2—C6	90.20 (14)	O3—C3—Fe1	178.3 (2)
C4—Fe2—C5	99.53 (13)	O4—C4—Fe2	178.2 (3)
C6—Fe2—C5	99.25 (14)	O5—C5—Fe2	178.3 (3)
C4—Fe2—S2	154.17 (9)	O6—C6—Fe2	177.4 (3)
C6—Fe2—S2	90.14 (10)	O7—C7—Fe3	179.4 (3)
C5—Fe2—S2	105.90 (10)	O8—C8—Fe3	179.2 (3)
C4—Fe2—S1	93.58 (10)	O9—C9—Fe3	176.3 (2)
C6—Fe2—S1	155.68 (10)	O10—C10—Fe4	177.3 (3)
C5—Fe2—S1	103.78 (11)	O11—C11—Fe4	177.5 (3)
S2—Fe2—S1	76.21 (3)	O12—C12—Fe4	177.7 (3)
C4—Fe2—Fe1	98.88 (10)	C14—C13—S1	112.8 (2)
C6—Fe2—Fe1	99.76 (10)	C14—C13—H13A	109.0
C5—Fe2—Fe1	153.37 (10)	S1—C13—H13A	109.0
S2—Fe2—Fe1	55.67 (3)	C14—C13—H13B	109.0
S1—Fe2—Fe1	55.92 (3)	S1—C13—H13B	109.0
C8—Fe3—C7	92.59 (14)	H13A—C13—H13B	107.8
C8—Fe3—C9	98.25 (14)	C15—C14—O13	110.4 (3)
C7—Fe3—C9	99.52 (13)	C15—C14—C13	131.7 (3)
C8—Fe3—S2	156.60 (10)	O13—C14—C13	117.7 (3)
C7—Fe3—S2	91.54 (10)	C14—C15—C16	106.7 (3)
C9—Fe3—S2	103.76 (10)	C14—C15—H15	126.7
C8—Fe3—S3	91.95 (10)	C16—C15—H15	126.7
C7—Fe3—S3	159.40 (10)	C17—C16—C15	106.8 (3)
C9—Fe3—S3	99.69 (10)	C17—C16—H16	126.6
S2—Fe3—S3	76.72 (4)	C15—C16—H16	126.6
C8—Fe3—Fe4	101.36 (10)	C16—C17—O13	110.2 (3)
C7—Fe3—Fe4	103.36 (10)	C16—C17—H17	124.9
C9—Fe3—Fe4	148.96 (10)	O13—C17—H17	124.9
S2—Fe3—Fe4	55.32 (3)	C19—C18—S3	109.1 (2)
S3—Fe3—Fe4	56.05 (3)	C19—C18—H18A	109.9
C12—Fe4—C11	92.05 (16)	S3—C18—H18A	109.9
C12—Fe4—C10	99.51 (15)	C19—C18—H18B	109.9
C11—Fe4—C10	99.31 (15)	S3—C18—H18B	109.9
C12—Fe4—S2	88.53 (10)	H18A—C18—H18B	108.3
C11—Fe4—S2	149.77 (11)	C20—C19—O14	110.0 (3)
C10—Fe4—S2	110.40 (11)	C20—C19—C18	133.3 (3)
C12—Fe4—S3	160.65 (10)	O14—C19—C18	116.7 (3)
C11—Fe4—S3	94.40 (11)	C19—C20—C21	106.7 (3)
C10—Fe4—S3	97.42 (11)	C19—C20—H20	126.6
S2—Fe4—S3	76.79 (4)	C21—C20—H20	126.6
C12—Fe4—Fe3	105.43 (11)	C22—C21—C20	106.4 (3)
C11—Fe4—Fe3	95.30 (11)	C22—C21—H21	126.8
C10—Fe4—Fe3	150.52 (10)	C20—C21—H21	126.8
S2—Fe4—Fe3	55.68 (3)	C21—C22—O14	111.2 (3)
S3—Fe4—Fe3	55.82 (3)	C21—C22—H22	124.4
C13—S1—Fe1	114.19 (11)	O14—C22—H22	124.4
			
C3—Fe1—Fe2—C4	−0.62 (13)	C2—Fe1—S2—Fe4	74.09 (11)
C1—Fe1—Fe2—C4	93.86 (14)	S1—Fe1—S2—Fe4	168.65 (5)
C2—Fe1—Fe2—C4	−128.58 (19)	Fe2—Fe1—S2—Fe4	−133.06 (6)
S2—Fe1—Fe2—C4	175.16 (10)	C3—Fe1—S2—Fe2	9.9 (2)
S1—Fe1—Fe2—C4	−88.32 (11)	C1—Fe1—S2—Fe2	105.66 (9)
C3—Fe1—Fe2—C6	−92.36 (14)	C2—Fe1—S2—Fe2	−152.84 (10)
C1—Fe1—Fe2—C6	2.12 (14)	S1—Fe1—S2—Fe2	−58.28 (3)
C2—Fe1—Fe2—C6	139.68 (19)	C3—Fe1—S2—Fe3	140.0 (2)
S2—Fe1—Fe2—C6	83.42 (11)	C1—Fe1—S2—Fe3	−124.24 (10)
S1—Fe1—Fe2—C6	179.94 (11)	C2—Fe1—S2—Fe3	−22.75 (10)
C3—Fe1—Fe2—C5	132.7 (2)	S1—Fe1—S2—Fe3	71.81 (5)
C1—Fe1—Fe2—C5	−132.9 (2)	Fe2—Fe1—S2—Fe3	130.10 (5)
C2—Fe1—Fe2—C5	4.7 (3)	C4—Fe2—S2—Fe4	123.2 (2)
S2—Fe1—Fe2—C5	−51.6 (2)	C6—Fe2—S2—Fe4	32.48 (11)
S1—Fe1—Fe2—C5	45.0 (2)	C5—Fe2—S2—Fe4	−67.18 (12)
C3—Fe1—Fe2—S2	−175.79 (9)	S1—Fe2—S2—Fe4	−167.85 (5)
C1—Fe1—Fe2—S2	−81.31 (10)	Fe1—Fe2—S2—Fe4	134.23 (6)
C2—Fe1—Fe2—S2	56.25 (17)	C4—Fe2—S2—Fe1	−11.0 (2)
S1—Fe1—Fe2—S2	96.52 (4)	C6—Fe2—S2—Fe1	−101.75 (11)
C3—Fe1—Fe2—S1	87.69 (9)	C5—Fe2—S2—Fe1	158.59 (10)
C1—Fe1—Fe2—S1	−177.82 (10)	S1—Fe2—S2—Fe1	57.92 (3)
C2—Fe1—Fe2—S1	−40.26 (17)	C4—Fe2—S2—Fe3	−131.5 (2)
S2—Fe1—Fe2—S1	−96.52 (4)	C6—Fe2—S2—Fe3	137.79 (11)
C8—Fe3—Fe4—C12	−100.70 (15)	C5—Fe2—S2—Fe3	38.12 (11)
C7—Fe3—Fe4—C12	−5.25 (15)	S1—Fe2—S2—Fe3	−62.54 (5)
C9—Fe3—Fe4—C12	131.2 (2)	Fe1—Fe2—S2—Fe3	−120.46 (5)
S2—Fe3—Fe4—C12	77.24 (12)	C8—Fe3—S2—Fe4	5.1 (3)
S3—Fe3—Fe4—C12	174.66 (12)	C7—Fe3—S2—Fe4	105.21 (9)
C8—Fe3—Fe4—C11	−7.10 (15)	C9—Fe3—S2—Fe4	−154.60 (9)
C7—Fe3—Fe4—C11	88.36 (15)	S3—Fe3—S2—Fe4	−57.69 (4)
C9—Fe3—Fe4—C11	−135.2 (2)	C8—Fe3—S2—Fe1	137.3 (2)
S2—Fe3—Fe4—C11	170.85 (12)	C7—Fe3—S2—Fe1	−122.56 (10)
S3—Fe3—Fe4—C11	−91.73 (12)	C9—Fe3—S2—Fe1	−22.37 (10)
C8—Fe3—Fe4—C10	112.5 (2)	S3—Fe3—S2—Fe1	74.54 (5)
C7—Fe3—Fe4—C10	−152.0 (2)	Fe4—Fe3—S2—Fe1	132.23 (5)
C9—Fe3—Fe4—C10	−15.6 (3)	C8—Fe3—S2—Fe2	−127.7 (2)
S2—Fe3—Fe4—C10	−69.5 (2)	C7—Fe3—S2—Fe2	−27.61 (10)
S3—Fe3—Fe4—C10	27.9 (2)	C9—Fe3—S2—Fe2	72.58 (10)
C8—Fe3—Fe4—S2	−177.94 (10)	S3—Fe3—S2—Fe2	169.49 (5)
C7—Fe3—Fe4—S2	−82.49 (10)	Fe4—Fe3—S2—Fe2	−132.83 (5)
C9—Fe3—Fe4—S2	53.92 (17)	C8—Fe3—S3—C18	5.48 (15)
S3—Fe3—Fe4—S2	97.42 (4)	C7—Fe3—S3—C18	108.1 (3)
C8—Fe3—Fe4—S3	84.64 (11)	C9—Fe3—S3—C18	−93.23 (14)
C7—Fe3—Fe4—S3	−179.91 (10)	S2—Fe3—S3—C18	164.79 (11)
C9—Fe3—Fe4—S3	−43.50 (17)	Fe4—Fe3—S3—C18	107.87 (11)
S2—Fe3—Fe4—S3	−97.42 (4)	C8—Fe3—S3—Fe4	−102.39 (10)
C3—Fe1—S1—C13	8.94 (13)	C7—Fe3—S3—Fe4	0.2 (3)
C1—Fe1—S1—C13	114.3 (3)	C9—Fe3—S3—Fe4	158.90 (10)
C2—Fe1—S1—C13	−92.07 (14)	S2—Fe3—S3—Fe4	56.91 (3)
S2—Fe1—S1—C13	165.90 (11)	C12—Fe4—S3—C18	−122.7 (4)
Fe2—Fe1—S1—C13	108.30 (11)	C11—Fe4—S3—C18	−13.60 (15)
C3—Fe1—S1—Fe2	−99.36 (9)	C10—Fe4—S3—C18	86.40 (15)
C1—Fe1—S1—Fe2	6.0 (3)	S2—Fe4—S3—C18	−164.30 (11)
C2—Fe1—S1—Fe2	159.63 (9)	Fe3—Fe4—S3—C18	−107.02 (11)
S2—Fe1—S1—Fe2	57.59 (4)	C12—Fe4—S3—Fe3	−15.7 (3)
C4—Fe2—S1—C13	−9.06 (14)	C11—Fe4—S3—Fe3	93.42 (11)
C6—Fe2—S1—C13	−107.5 (3)	C10—Fe4—S3—Fe3	−166.57 (11)
C5—Fe2—S1—C13	91.67 (15)	S2—Fe4—S3—Fe3	−57.27 (3)
S2—Fe2—S1—C13	−165.02 (11)	Fe1—S1—C13—C14	68.5 (3)
Fe1—Fe2—S1—C13	−107.36 (12)	Fe2—S1—C13—C14	144.1 (2)
C4—Fe2—S1—Fe1	98.30 (10)	C17—O13—C14—C15	0.2 (3)
C6—Fe2—S1—Fe1	−0.1 (3)	C17—O13—C14—C13	175.9 (2)
C5—Fe2—S1—Fe1	−160.97 (10)	S1—C13—C14—C15	−130.0 (3)
S2—Fe2—S1—Fe1	−57.66 (3)	S1—C13—C14—O13	55.3 (3)
C12—Fe4—S2—Fe1	129.09 (13)	O13—C14—C15—C16	0.3 (3)
C11—Fe4—S2—Fe1	−139.4 (2)	C13—C14—C15—C16	−174.7 (3)
C10—Fe4—S2—Fe1	29.50 (12)	C14—C15—C16—C17	−0.6 (3)
S3—Fe4—S2—Fe1	−63.61 (6)	C15—C16—C17—O13	0.7 (3)
Fe3—Fe4—S2—Fe1	−121.04 (6)	C14—O13—C17—C16	−0.6 (3)
C12—Fe4—S2—Fe2	22.51 (13)	Fe3—S3—C18—C19	−178.94 (18)
C11—Fe4—S2—Fe2	114.0 (2)	Fe4—S3—C18—C19	−103.4 (2)
C10—Fe4—S2—Fe2	−77.09 (12)	C22—O14—C19—C20	−1.3 (4)
S3—Fe4—S2—Fe2	−170.20 (5)	C22—O14—C19—C18	−179.5 (3)
Fe3—Fe4—S2—Fe2	132.38 (6)	S3—C18—C19—C20	−105.8 (4)
C12—Fe4—S2—Fe3	−109.87 (12)	S3—C18—C19—O14	71.9 (3)
C11—Fe4—S2—Fe3	−18.3 (2)	O14—C19—C20—C21	1.1 (4)
C10—Fe4—S2—Fe3	150.53 (11)	C18—C19—C20—C21	178.9 (3)
S3—Fe4—S2—Fe3	57.42 (3)	C19—C20—C21—C22	−0.5 (4)
C3—Fe1—S2—Fe4	−123.2 (2)	C20—C21—C22—O14	−0.4 (4)
C1—Fe1—S2—Fe4	−27.40 (10)	C19—O14—C22—C21	1.0 (4)

## References

[bb1] Bruker (1999). *SMART* and *SAINT* Bruker AXS Inc., Madison, Wisconsin, USA.

[bb2] Coleman, J. M., Wojcicki, A., Pollick, P. J. & Dahl, L. F. (1967). *Inorg. Chem.* **6**, 1236–1242.

[bb3] Shaver, A., Fitzpatrick, P. J., Steliou, K. & Butler, I. S. (1979). *J. Am. Chem. Soc.* **101**, 1313–1315.

[bb4] Sheldrick, G. M. (1996). *SADABS* University of Göttingen, Germany.

[bb5] Sheldrick, G. M. (2008). *Acta Cryst.* A**64**, 112–122. 10.1107/S0108767307043930 18156677

[bb6] Song, L.-C., Hu, Q.-M., Jia, G.-F. & Wang, J.-Y. (1992). *Sci. China (Ser. B)*, **35**, 1–9.

[bb7] Song, L.-C., Hu, Q.-M., Zhang, L.-Y., Wang, H., Zhou, Z.-Y. & Liu, L. (1991). *J. Organomet. Chem.* **412**, C19–C22.

[bb8] Song, L.-C., Kakiata, M., Wang, J.-T., Wang, R.-J. & Wang, H.-G. (1988). *J. Organomet. Chem.* **340**, 239–248.

